# Mapping the Ulcerative Colitis Patient Journey in Saudi Arabia from Healthcare Professionals’ Perspective: A Cross-Sectional Non-Interventional Study

**DOI:** 10.3390/jcm14051621

**Published:** 2025-02-27

**Authors:** Nahla Azzam, Mahmoud Mosli, Abdulelah Almutairdi, Mesfer Alghamdi, Yaser Meeralam, Khalid Alolimi, Mohammad Arsalan, Badr Al-Bawardy

**Affiliations:** 1 Division of Gastroenterology, Department of Medicine, King Saud University, Riyadh 11451, Saudi Arabia; 2 Division of Gastroenterology, Department of Medicine, King Abdul-Aziz University, Jeddah 21589, Saudi Arabia; mmosli@kau.edu.sa; 3 Division of Gastroenterology, Department of Medicine, King Faisal Specialist Hospital and Research Center, Riyadh 12271, Saudi Arabia; aalmutairdi@kfshrc.edu.sa (A.A.); balbawardy@kfshrc.edu.sa (B.A.-B.); 4 College of Medicine, Alfaisal University, Riyadh 11533, Saudi Arabia; 5 Internal Medicine Department, AlHada Armed Force Hospital, Taif 26792, Saudi Arabia; mnzagh@yahoo.com; 6 Digestive and Liver Health and Advanced Endoscopy Center, King Abdullah Medical City, Makkah 24246, Saudi Arabia; meeralam@kamc.med.sa; 7 Pfizer, Medical Affairs, Riyadh 13519, Saudi Arabia; khalid.alolimi@pfizer.com (K.A.); mohammadsaeed.arsalan@pfizer.com (M.A.); 8 Section of Digestive Diseases, Department of Internal Medicine, Yale School of Medicine, New Haven, CT 63104, USA

**Keywords:** ulcerative colitis, patient journey, Saudi Arabia

## Abstract

**Background/Objectives**: The burden of ulcerative colitis (UC) is increasing in Saudi Arabia (KSA), and patients with UC often suffer from delays in diagnosis and appropriate management. This study investigates the current UC patient journey in KSA from the healthcare professionals’ (HCPs) perspective. It aims to evaluate treatment patterns, identify critical gaps, and provide insights to guide interventions that enhance the quality of life for UC patients in KSA. **Methods**: Quantitative interviews were conducted with 60 HCPs (45 gastroenterologists and 15 internal medicine specialists) from different regions in Saudi Arabia (KSA) using a Computer-Assisted Personal Interview (CAPI) system. The survey domains included clinical symptoms, diagnostic testing, endoscopic scoring, treatment goals, and medication sequencing. **Results**: Data were collected from 60 HCPs with an average of 17 ± 12.5 years of experience. Most patients with UC were referred by general practitioners (28%), internal medicine physicians (25%), followed by surgeons (16%). The first-ranked treatment goals were clinical remission (53.3%), endoscopic remission (35%), and improvement of quality of life (33.3%). For outpatient moderate-to-severe UC, the most common first-line treatments are oral systemic steroids (34%), 5-aminosalicylates (5-ASAs) (26%), and TNF-α inhibitors (21%). While second-line treatment rankings were TNF-α inhibitors (23%), followed by Interleukin 12/23 inhibitors (19%), and Janus kinase (JAK) inhibitors (14%). Sphingosine 1-phosphate (S1P) receptor modulators are not well-utilized due to a lack of availability (88%), unfamiliarity with the treatment (24%), and formulary exclusion (12%). **Conclusions**: In conclusion, most UC patients are referred by general practitioners. Treating gastroenterologists prioritize clinical remission as a treatment goal. Corticosteroids remain overutilized as reflected by treating physicians’ responses. The underutilization of advanced therapies underscores the need for enhanced education and improved access to integrate emerging therapies effectively.

## 1. Introduction

Ulcerative Colitis (UC) is an idiopathic, chronically relapsing inflammatory bowel disease (IBD) marked by continuous inflammation of the colonic mucosa, beginning in the rectum and typically spreading proximally throughout the entire colon [1,2].

UC is characterized by colonocyte-associated defects, dysbiosis, increased expression of Toll-like receptors, neutrophils, and IgG1 antibodies. Furthermore, up to 15% of individuals may initially present with severe disease [3]. Rectal bleeding, abdominal pain, and diarrhea are the predominant symptoms [4]. An accurate diagnosis of UC necessitates an integrative approach comprising several diagnostic tools and clinical parameters [5].

Treatment strategies have evolved into a “treat-to-target approach” supported by head-to-head comparative trials to facilitate evidence-based decision-making [6].

Typically, the severity of the disease plays a crucial role in selecting appropriate treatment strategies [7]. Novel therapeutic options encompass antibodies targeting interleukin-23, selective inhibitors of Janus kinase (JAK), and sphingosine-1-phosphate receptor (S1PR) modulators.

In 2017, the global number of (IBD) cases reached 6.8 million. Regions with a high Sociodemographic Index (SDI) demonstrated the highest age-adjusted prevalence rates, whereas areas with a low SDI had lower prevalence rates [8]. New cases are still on the rise, presenting ongoing challenges for HCPs who face a longer duration of the disease accompanied by treatment-related complications such as infections and malignancies [9].

As for Saudi Arabia (KSA), several epidemiological studies have also been carried out to determine UC prevalence and incidence rates among adults in different regions. The estimated incidence rate was 0.94 cases per 100,000 people [10].

Most patients (92%) in Saudi Arabia received a diagnosis in less than 6 months, while 77% were diagnosed in less than one month, as shown by three reports from the Middle East. These reports disclosed that there was a delay in the diagnosis of patients from the time they started experiencing the symptoms of UC. However, the process was comparatively accelerated compared to other nations, such as Oman and Iran [11].

Limited research has been conducted to comprehend the UC patient journey from the HCPs’ perspective and to elucidate the primary unmet needs associated with this disorder. Patients who have cycled through multiple treatment regimens expressed concern regarding the potential exhaustion of therapeutic options, potentially leading to surgical intervention.

This study was designed to map the UC journey in Saudi Arabia (KSA) from the perspective of healthcare professionals (HCPs) and identify key challenges. A steering committee carefully designed the study’s questionnaire and methodology to meet the research objectives. Additional objectives include assessing physicians’ awareness and attitudes toward novel therapeutic agents, determining drivers and barriers for transitioning to alternative classes, examining treatment and referral patterns, and identifying market gaps in the UC therapeutic landscape.

## 2. Materials and Methods

### 2.1. Study Design

This cross-sectional, exploratory, and descriptive study uses a quantitative interview-based approach. Interviews were conducted with 60 healthcare physicians (HCPs), including gastroenterologists and internal medicine specialists from different geographical areas in Saudi Arabia (KSA). A quantitative questionnaire designed for this study was completed in English using a Computer Assisted Personal Interview (CAPI) system during the interview with each doctor (See Supplementary material, “UC Journey of HCPs at KSA Questionnaire”) This study did not involve patients directly. The sampling process was conducted randomly. The inclusion criteria for physicians’ participation in the questionnaire were carefully designed to minimize potential bias. The selection of the participants was based on predefined, objective factors such as their specialty, years of experience, geographic location, and involvement in relevant clinical practices. Additionally, we ensured a diverse and representative sample that helped in reducing the selection bias.

### 2.2. Physician Questionnaire Will Include but Is Not Limited to

(A) Workload and Patient Profiling: Including the most common symptoms that UC patients experience, categories of UC patients, scoring systems used to categorize UC patients, and the percentages of controlled/uncontrolled and Naïve/referred patients.
(B) Diagnosis: Including methods of screening and confirming the diagnosis of UC.
(C) Treatment: Including treatment goals, barriers that prevent diagnosed UC patients from receiving treatment, guidelines incorporated into practice, most prescribed treatment classes, the percentage distribution of UC patients that are treated with different molecules, the duration on which patients stay on the same treatment, patient compliance, criteria considered before stopping an on-going treatment or switching to another treatment, factors affecting treatment decisions, attributes of different treatments, whether there are specific treatments requested by UC patients, and unmet needs for the currently available treatments for UC.
(D) Target Molecule Profile Testing: Including the HCP’s first reaction regarding the molecule profile, how it is compared to other molecules available in the market for UC in KSA, the patient profile considered most appropriate for this molecule, his willingness and rationale behind trying this new molecule in his practice if it was included in the guidelines as a treatment option, and his willingness to consider oral treatment for moderate or severe UC patients in his clinical practice.

### 2.3. Survey Development

The survey was conducted based on:

1. Literature Review: A thorough review of existing studies, guidelines, and expert opinions related to UC care.
2. Expert Input: A panel of IBD experts contributed to designing survey items to ensure relevance and comprehensiveness.
3. Domains: The survey covered key aspects of the UC journey, including:
  - Initial presentation and diagnostic challenges.
  - Treatment approaches and decision-making processes.
  - Use of advanced therapies and surgical interventions.
  - Perceived barriers in delivering optimal care.

### 2.4. Validation of Survey Content

To ensure the validity of the survey:

1. Face Validity: The survey was reviewed by a panel of 6 IBD experts to assess clarity, relevance, and comprehensiveness.
2. Content Validity: Items were mapped to predefined domains and evaluated using a Content Validity Index (CVI). Items with a CVI > 0.8 were retained.
3. Pilot Testing: The survey was piloted to identify ambiguities and assess reliability. Adjustments were made based on their feedback.

### 2.5. Study Size

Quantitative interviews were conducted with a mix of 60 HCPs (45 gastroenterologists and 15 internal medicine specialists) in KSA to understand the patient journey, diagnostic modalities, disease monitoring, treatment preferences, and sequencing using a CAPI system with each doctor.

Participants’ Selection Criteria:

- Working in the KSA healthcare system.
- Specialty: Gastroenterology (*n* = 45) and Internal Medicine (*n* = 15).
- Years of experience: 3–30 years in Gastroenterology or Internal Medicine.
- Primary Practice Sector: Institutional Hospital (Public) [MOH/TPH] (85%) and Private Hospital (15%).
- Patient Engagement: Over 50% of their professional time is dedicated to direct patient care.
- Patient Interaction: Engage with over 50 patients on an average monthly basis.
- UC Patient Interaction: See/manage a minimum of 10 UC patients monthly.
- UC Patient Severity: At least 30% of monthly UC patients should be of moderate to severe intensity.

### 2.6. Survey Settings

Interviews were conducted with HCPs using a CAPI system with each doctor, either in the form of face-to-face interviews (a member of the field team visited the HCP in the hospital and conducted an interview with them in English using the CAPI system) or via online platforms (Microsoft Teams and Zoom).

### 2.7. Data Source/Data Collection

Before data collection, local fieldwork partners identified appropriate HCPs according to the inclusion criteria. HCPs were provided with an overview of the survey. Ultimately, all participating HCPs (gastroenterologists and internal medicine specialists) were interviewed through a CAPI system. The resulting data were used to map the UC patient journey care pathway and identify key challenges.

### 2.8. Data Quality

To ensure the validity of the results, the data collected must be of good quality. This implies minimizing missing data and ensuring the collected data are accurate. To maximize data quality, standardized instructions on survey design, methodology, and procedures were provided:

(a) HCPs were provided with an overview of the survey.
(b) Any electronic materials for HCPs were tested thoroughly to ensure that all questions appear correctly on screen, allow easy interpretation/completion, and that all routing and logic checks work correctly.
(c) All electronic materials include pre-programmed data checks, which queried unfeasible data.
(d) First, a pilot phase was conducted on 10% of the sample as a quality measure to ensure the validity of the research tools and assess the flow and comprehension of the survey.

### 2.9. Statistical Analysis

Analyses were conducted using Dimensions software 6.0.1 (IBM SPSS Base Professional for survey scripting and survey reporter for analysis) [12]. Variables with missing responses were excluded from the analysis. For this reason, we would expect the denominator of the number of responses to vary from variable to variable. Stata installation tools, as provided by the associated software vendor, were used to validate the installation of the software. This validation occurred on every new installation or version update of a statistical software package. This validation must also run if the operating system, or if the computer themselves, were changed/updated.

### 2.10. Data Analysis

The Ipsos statistician team performed all analyses. This is an exploratory survey, and the analyses were primarily descriptive in nature; no formal hypothesis was tested. Descriptive statistics that were shown depend on the type of variable being described:

1. Numeric: the number of non-missing values, mean, standard deviations, minimum, median, quartiles, and maximum.
2. Categorical: the number and percentage of subjects in each category.

### 2.11. Ethical Approval

It was reviewed and approved by the King Saud University Medical City (KSUMC) Institutional Review Board (IRB), ensuring ethical data collection and management practices.

## 3. Results

A total of 60 HCPs (45 gastroenterologists and 15 internal medicine specialists) based in Jeddah (*n* = 31; 52%), Riyadh (*n* = 22; 37%), and Dammam (*n* = 7; 11%) participated in the questionnaire throughout the period from 28 May 2024 to 17 July 2024. The vast majority are working in the public sector (*n* = 50; 83%) with an average of 17 ± 12.5 years of experience. Specifically, 16% of participants had 6–10 years of experience, 32% had 11–15 years, and 52% had 16–30 years.

UC patients represented 11% (*n* = 30) of their monthly patient load. Almost 50% of patients seen by respondents have moderate to severe or acute severe UC. Of these patients, 78% had controlled disease per physician responses. Regarding patients’ referral patterns, 57% were referred from different specialties, mainly general practitioners (28%), internal medicine physicians (25%), and surgeons (16%). Respondents reported that UC patients’ initial symptoms are chronic diarrhea (92%), rectal bleeding (92%), and abdominal pain (88%). HCPs stated that clinical remission (*n* = 32; 53.3%), endoscopic remission (*n* = 21; 35%), and quality of life improvement (*n* = 20; 33.3%) are the most important treatment goals.

A total of 60% of physicians in Saudi Arabia (KSA) utilize the Modified Mayo System (MMS) in clinical scoring, while for endoscopic scoring of the UC patients, 84% of GIs use the Mayo Score/Disease Activity Index (DAI). In addition, diverse methods are used to screen for UC, mainly biomarkers and stool samples, but UC is ultimately confirmed by colonoscopy/sigmoidoscopy, with biopsy in almost all cases (Table 1).

HCPs rely primarily on guidelines from the European Crohn’s and Colitis Organization (ECCO) (33%), the Saudi Gastroenterology Association (SGA) (24%), and the American Gastroenterological Association (AGA) (23%) in their practice for treating UC. In contrast, internal institution guidelines and their practice appear to have less influence. The treatment algorithm most frequently used was oral systemic steroids as the first line, TNF-α inhibitors as the second line, and JAK inhibitors as the third line, employed by 15% of HCPs in patients with moderate-to-severe UC and 22% in acute severe UC.

Gastroenterologists had diverse treatment choices for moderate-to-severe UC, but oral systemic steroids and 5-ASA were the most common first-line treatments, accounting for nearly 60% of the choices. TNF-α inhibitors were preferred for the second line, followed by interleukin 12/23 inhibitors. In the third line, TNF-α and JAK inhibitors were most common.

**Figure 1:**
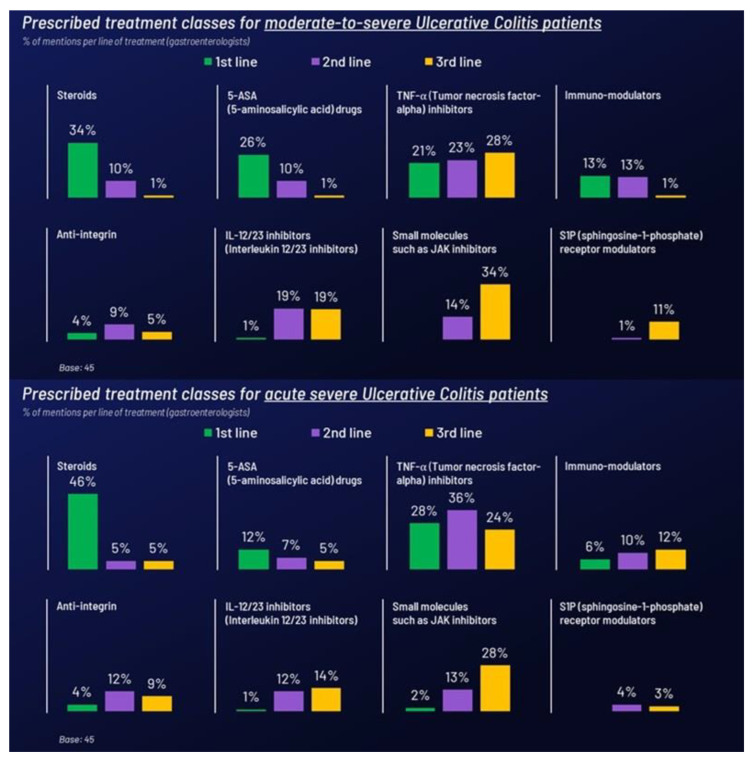
Most prescribed treatment classes for UC as first-, second-, and third-line therapy.

Generally, treatment choices for acute severe UC were like those for moderate cases, although 5-ASA was less likely to be placed as the first line. Oral systemic steroids were the predominant first-line treatment, used about half the time, with TNF-α inhibitors a distant second position. However, TNF-α inhibitors were favored by around 36% of GIs for second-line treatment, with other choices varying widely. In the third line, JAK inhibitors slightly edged out TNF-α inhibitors by just four percentage points (Figure 1). Interestingly, 90% of HCPs believe that treatment choices differ in the case of pregnancy with moderate to severe UC. TNF-α inhibitors were the top choice by 43% of HCPs due to their safety profile, followed by oral systemic steroids by 30%.

**Figure 2:**
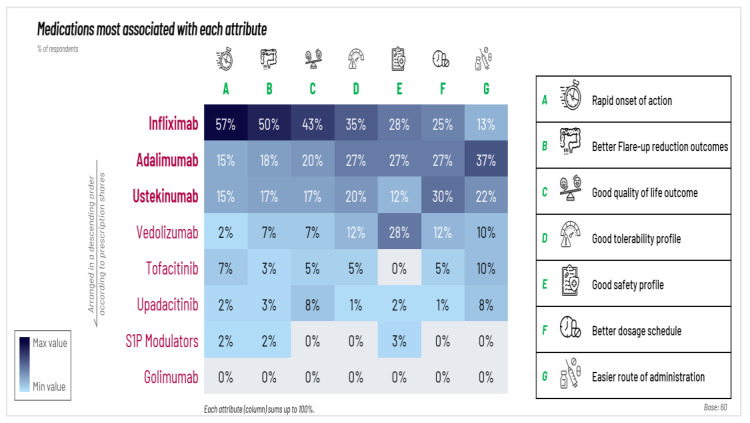
Medications most commonly associated with certain attributes.

HCPs were asked to choose medications most associated with certain attributes such as rapid onset of action, better flare-up reduction outcomes, and good quality of life. Infliximab is the top choice for its rapid onset of action, superior flare-up reduction, and significant improvement in quality of life. However, regarding safety, infliximab, adalimumab, and vedolizumab were all ranked similarly as highest (Figure 2).

**Figure 3:**
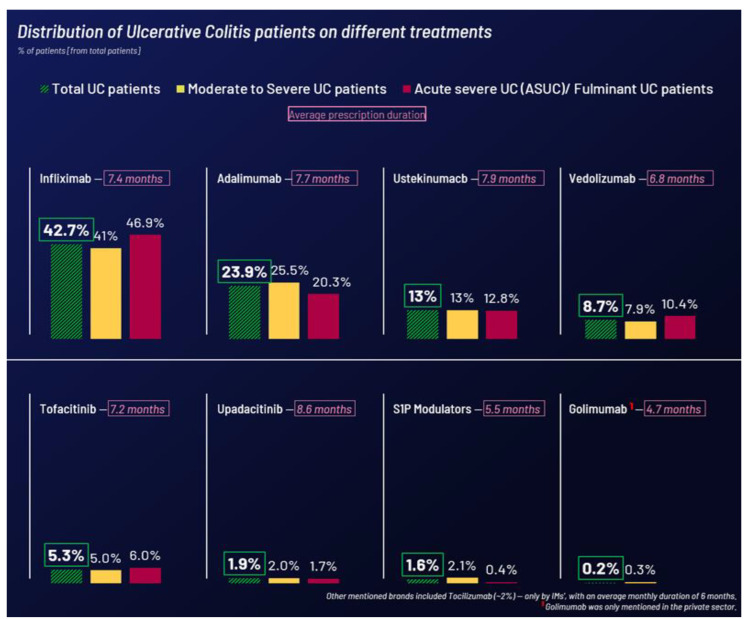
Most prescribed treatment classes for moderate UC and Acute severe/fulminant UC (ASUC).

As shown in Figure 3, infliximab was prescribed to nearly 43% of all UC patients (moderate to severe or acute severe), followed by adalimumab at 24%. Ustekinumab and vedolizumab are utilized less frequently. HCP reports that around 80% of patients fully comply with biologic and small-molecule treatments. Decisions to stop or switch treatments are mainly made when symptomatic or clinical remission is not achieved.

**Figure 4:**
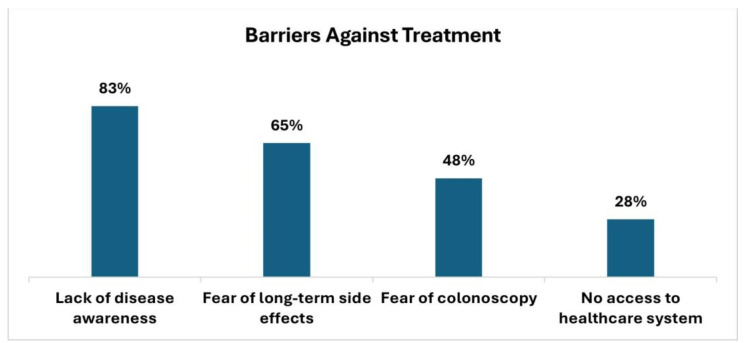
Patients’ barriers against UC treatment from HCPs’ perspective.

Barriers against treatment initiation are shown in (Figure 4), HCPs in Saudi Arabia (KSA) believe that patients’ lack of disease awareness (83%), fear of long-term treatment side effects (65%), and anxiety about undergoing colonoscopies (48%) lead to delayed doctor visits. Additionally, limited access to healthcare systems in the public sector (28%) is a significant issue.

HCPs were queried regarding obstacles to prescribing S1P receptor modulators. Lack of availability (88%) was the most substantial barrier, followed by unfamiliarity (24%), formulary exclusion (12%), and insufficient supporting research (8%).

## 4. Discussion

Our findings reveal that a significant proportion (nearly 50%) of UC patients presents with moderate-to-severe or acutely severe disease. The referral patterns indicate that a substantial proportion (57%) originates from other specialties, primarily general practitioners and internal medicine, highlighting the importance of interdisciplinary collaboration in UC care and facilitating referral pathways to GI.

The majority of HCPs utilized standard scoring systems for clinical and endoscopic assessment, such as the Modified Mayo Score (MMS) and the Mayo endoscopic sub-score. This demonstrates a thorough understanding of standardized UC monitoring and disease assessment by HCP in KSA. The wide range of screening techniques used, including biomarkers and stool samples, followed by colonoscopy/sigmoidoscopy with biopsy as a gold-standard approach to UC, elucidates a holistic assessment of UC. This is consistent with Saudi Gastroenterology Association (SGA) guidelines [10].

A key finding from this study indicates that HCPs demonstrate greater confidence in TNF-α inhibitors, 5-aminosalicylates (5-ASAs), and oral systemic steroids as first-line treatments compared to other drug classes. This highlights the limitation of HCP practice patterns, as per recent guidelines, including the ECCO (European Crohn’s and Colitis Organization), Saudi Arabia consensus guidelines for the management of IBD, and AGA (American Gastroenterological Association) guidelines. Corticosteroid utilization should be limited [7,10,13]. This is particularly relevant given that a majority (64%) of their patients (who ask for a specific administration route) prefer oral drugs which may shift in prescribing patterns toward newer oral therapies, as second-line treatments. However, adopting these newer therapies hinges on their inclusion in treatment guidelines and hospital formularies.

Moreover, HCPs reported reliance on established guidelines such as ECCO and AGA, while internal institutional guidelines appeared to have less influence on their practice. This behavior is reassuring, as it promotes adherence to evidence-based recommendations and may contribute to more consistent and effective patient care.

Regarding the treatment algorithm, this sequential approach, starting with oral systemic steroids, then TNF-α inhibitors, and finally JAK inhibitors, reflects a step-up strategy based on disease severity, response to prior therapies, and balancing efficacy with safety considerations. The diversity of treatment choices, particularly in moderate-to-severe UC, where oral systemic steroids and 5-ASAs dominate first-line therapy, underscores the individualized nature of UC management. HCPs agree upon this personalized approach since most of them stated that treatment strategy differs in the case of pregnancy.

Additionally, due to lack of availability as it is not yet Saudi Food and Drug Administration (FDA) approved and recent introduction to clinical practice, the limited use of S1P receptor modulators, particularly in first- and second-line treatment, necessitates determining the optimal place of S1P receptor modulators within the broader treatment strategy for UC. This includes comparing them to existing therapies like biologics and JAK inhibitors through head-to-head trials, exploring their potential in combination therapies, and determining whether they are more effective as first-, second-, or third-line treatments. Studies on cost-effectiveness and patient-reported outcomes, such as quality of life and symptomatic remission, are also important for understanding the overall benefit to patients.

The finding that infliximab is favored for rapid onset of action, flare-up reduction, and quality of life improvement aligns with existing publications supporting its efficacy in UC [14]. While the “VARSITY trial” provides clear evidence of vedolizumab’s superior efficacy over adalimumab, the fact that respondents ranked both agents similarly suggests a potential gap between evidence and clinical practice [15]. This highlights the need for continued education and dissemination of the latest research findings to ensure evidence-based positioning of these therapies in UC management. The preference for adalimumab for ease of administration, likely due to its subcutaneous administration compared to other drugs, emphasizes the importance of patient convenience and adherence in treatment selection and facilitates the inclusion of oral therapies despite HCPs’ hesitation to prescribe them as a first-line therapy.

A comparable investigation in Canada and other European countries by Schreiber et al., which examined the perspectives of UC patients and HCPs and identified gaps in UC management, revealed that despite patients and HCPs being satisfied with the predominant treatment (5-ASA), HCPs often underestimated disease severity and some symptoms experienced by patients, such as flares. This finding confirms the importance of conducting an additional study in Saudi Arabia (KSA) that focuses on the patient’s viewpoint. Such research would help uncover hidden gaps and enhance communication between patients and HCPs throughout the UC treatment journey, ultimately leading to improved outcomes [16].

Furthermore, reports from a global survey for more than 6 months across 10 countries (including the United States, France, and Japan), indicated that the emotional impact of UC was insufficiently prioritized during HCPs’ discussions with patients [17]. Consequently, a critical component of future research should focus on elucidating the key factors contributing to this discrepancy.

Comprehensive educational initiatives and patient support programs are valuable in tackling patients’ fear of colonoscopy and long-term side effects, as well as their lack of awareness about the disease. Moreover, promoting shared decision-making between patients and HCPs, empowering patients to participate actively in their treatment journey, and fostering a stronger therapeutic alliance will improve patient adherence to treatment plans.

Conducting a segmentation study is very important to classify doctors based on their prescribing profiles and the factors influencing their decisions. This will allow us to communicate tailored messages accordingly and help provide personalized treatment plans based on patients’ profiles and characteristics.

There is currently a knowledge gap that this survey intends to address concerning the UC patient journey care pathway in Saudi Arabia (KSA). The key strength of this survey is, that it is the first of its kind from Saudi Arabia or the Middle East. In addition, it provided valuable real-world insights from the perspective of HCPs to understand this journey better, along with key challenges encountered by patients throughout their journey. However, as with all surveys of this type, there were limitations. First, the survey caused an administrative burden on participants. To reduce this burden, the materials were as short and user-friendly as possible. Another limitation of this survey was that it was the only representative of HCPs willing to participate and did not capture the perceptions and unmet needs of those not willing to participate. Furthermore, the generalizability of the results was also limited by the small sample size of this study; only 60 HCPs (45 gastroenterologists and 15 internal medicine specialists). Therefore, a larger sample size from different regions is required to better understand its impact on practice patterns.

## 5. Conclusions

Our study is the first to explore the ulcerative colitis (UC) journey in Saudi Arabia (KSA) from the perspective of healthcare professionals (HCPs), identifying unmet needs in patient care. While diagnosis generally adhered to global and national guidelines, treatment algorithms varied and often lacked a personalized approach. Oral systemic steroids and TNF-α inhibitors were the most trusted first- and second-line therapies, while newer oral treatments (e.g., JAK inhibitors and S1P receptor modulators) were underutilized despite patient preferences for oral options. Limited patient awareness highlights the need for educational initiatives. Key treatment goals remain clinical remission, endoscopic remission, and improved quality of life.

## Figures and Tables

**Table 1:** Scoring System and Diagnostic Tools.

Scoring System			
**Clinical Scoring System Utilized**	**% HCPs**	**Endoscopic Scoring System Utilized**	**% GIs**
Modified Mayo Score (MMS)	60	Mayo Endoscopic Sub-Score	84
The Truelove and Witts	37	Ulcerative Colitis Endoscopic Index of Severity (UCEIS)	24
Simple Clinical Colitis Activity Index (SCCAI)	25		
**Diagnostic Tools**			
**Screening Methods**	**% HCPs**	**Confirmation Methods**	**% GIs**
Biomarkers	85	Colonoscopy/Sigmoidoscopy with Biopsy	96
Stool Sample	78	Colonoscopy/Sigmoidoscopy	24
Lab Tests	78		
X-Ray or CT-Scan	53	X-Ray or CT-Scan	2
Magnetic resonance Enterography (MRE)	27		

## Data Availability

The following supporting information “UC Journey of HCPs at KSA Questionnaire” can be downloaded as a supplementary material, and the created questionnaire presented in this study are available on reasonable request from the corresponding author.

## Supplementary Materials

The following supporting information can be downloaded at: https://www.mdpi.com/article/10.3390/jcm14051621/s1.
