# Supplementary material for: Mapping the Ulcerative Colitis Patient Journey in Saudi Arabia from Healthcare Professionals’ Perspective: A Cross-Sectional Non-Interventional Study
Source: J Clin Med. 2025 Feb 27;14(5):1621. doi: 10.3390/jcm14051621 (PMC11899997; doi:10.3390/jcm14051621)
Supplement: Supplementary file 1 [file jcm-14-01621-s001.zip › jcm-3444687-supplementary.pdf]

Ulcerative Colitis Journey in KSA
HCP Questionnaire

Sample quota: **60** HCPs (45 Gastros and 15 IMs)
Methodology: **CAPI**
Interview length: **30** minutes

PRE-INTRO QUESTION
What is your interviewer name / ID?

INTRODUCTION:
This survey is being conducted by Ipsos; an independent market research agency (headquartered in France) on behalf of a pharmaceutical company.
We are conducting research about **Ulcerative Colitis** and would like to ask you some questions on this topic. The survey will take around **30** minutes of your time.

With your consent, your information will only be collected and used for market research and analysis.
Any information you give will be treated in the strictest confidence and results will only be reported back on an aggregated basis.
As a member of EphMRA, Ipsos is bound by EphMRA Code of Conduct and all applicable laws protecting your personal data and responses. The study is conducted in compliance with ESOMAR guidelines.
You have the right to withdraw from the interview at any time. For more information about your rights please see our privacy notice, it is available at *https://www.ipsos.com/en/ipsos-privacy-notice*.
Any information you give will be treated in the strictest confidence and results will only be reported back on an aggregated basis. You will receive an honorarium for your participation.

____

[AE DISCLAIMER]
We are required to pass on to our client details of adverse events/product complaints pertaining to their products that are mentioned during the interview. If this happens, we will need to collect details and report the event, even if you have already done so

You will be asked whether you consent to assist by providing additional information to the client company's drug safety department for their follow up, but you may choose to remain anonymous. This will have no impact on the confidentiality and anonymity associated with the interview itself.

Please confirm that:

☐ I have read, understood and accept the points above and am happy to proceed with the market research survey on those basis **[MANDATORY TO CONTINUE]**

☐ No, I do not want to participate in this research [CLOSE]

SCREENER

To check your eligibility for the study, we would like to ask you a few initial questions.

S1. Which of the following best describes your primary specialty?

Single answer
All respondents

Code #	Code Label	Scripting Instruction
1	Gastroenterology	Continue (Quota n=45)
2	Internal Medicine (Gastro)	Continue (Quota n=15)
99	Other	**Terminate**

S2. For how many years have you practiced as a qualified medical professional in the field of Gastroenterology?

Code #	Code Label	Mark (SA)
1	**Less than 3 years**	**Terminate**
2	**3 – 5** years	Continue
3	**6 – 10** years	Continue
4	**11 – 15** years	Continue
5	**16 – 20** years	Continue
6	**21 – 30** years	Continue
7	More than 30 years	**Terminate**

S3. What is the type of institution you primarily practice at?
Single answer
All respondents

Code #	Code Label	Scripting Instruction
1	Institutional Hospital (Public) [MOH/TPH]	Continue - n=85% for each specialty (Gastro and IM)
2	Private Hospital	Continue - n=15% for each specialty (Gastro and IM)

S4. In a typical month, approximately what proportion of your time is spent <u>in direct patient care</u>?

Numeric entry
All respondents

#	Code Label	% of time
1	% of your professional time in direct patient care	………

MIN: 0
MAX: 100
Terminate if <50%

S5. Could you please provide an approximate number of patients whom you see/manage in an average month?
<u>Please count a patient only once if they make repeat visits during the month</u>

#	Code Label	No. of patients
1	Approx. no. of patients seen/managed in an average month	………

Terminate if <50 patients per month
MIN: 0
MAX: 500

S6. Out of these [insert S5. answer] patients, what is the approximate number of <u>Ulcerative Colitis</u> (UC) patients you see/manage in an average month? This includes either new or follow-up patients

#	Code Label	No. of UC patients
1	Approx. no. of **<u>Ulcerative Colitis</u>** patients seen/managed in an average month	………

Terminate if <10 UC patients per month
MIN: 0
MAX: S5. answer

S7. Please split the [insert S6. answer] Ulcerative Colitis patients you manage in a month to the following categories:

#	Code Label	No. of UC patients	Scripting Instruction
1	Mild Ulcerative Colitis	………	Continue
2	Moderate Ulcerative Colitis	………	Terminate if sum of (code 2 + code 3) is less than **30%** of S6 answer
3	Severe Ulcerative Colitis	………	

End of Screener

Main Questionnaire

Thank you for completing those questions. We are now going to ask you some questions regarding your UC patients and your prescriptions and/or recommendations.

A) Workload & Patient Profiling

Q1. Among the [insert S6. answer] Ulcerative Colitis (UC) patients you manage in a month, what are the most common symptoms of Ulcerative Colitis they experience?

All respondents
Multiple answers
Randomize statements

Q2. From all the symptoms mentioned, please rank the symptoms that makes the patient to seek doctor's help.
Rank up to 3 ranks, in which rank 1 means the most common symptom for which patient seeks doctor's help.

Maximum 3 choices
Randomize statements
Show only the codes selected in Q1

#	Code Label	Q1.	Q2.

		Commonly experienced symptoms (MA)	Rank TOP 3 symptoms for which patient seeks doctor's help
1	Chronic diarrhoea	☐	1,2,3
2	Rectal bleeding	☐	1,2,3
3	Abdominal pain and cramping	☐	1,2,3
4	Rectal pain	☐	1,2,3
5	Urgency to defecate	☐	1,2,3
6	Tenesmus	☐	1,2,3
7	Fatigue	☐	1,2,3
8	Fever	☐	1,2,3
10	Others, please specify	☐	1,2,3

Q3. Please indicate the scoring systems you use the <u>most</u> for categorization of your Ulcerative Colitis patients:

- **a. Clinical scoring systems**
- **b. Endoscopic scoring systems**

All respondents

#	Code Label	Clinical scoring systems (MA)	Endoscopic scoring systems (MA)
1	Mayo score / Disease Activity Index (DAI)		☐
2	Modified Mayo Score (MMS)	☐	
3	Simple Clinical Colitis Activity Index (SCCAI)	☐	
4	The Truelove and Witts	☐	
5	Ulcerative Colitis Endoscopic Index of Severity (UCEIS)		☐
6	None, I do not use a scoring system	○	○
7	Others, please specify	☐	☐

Q4. Among the [insert S6. answer] Ulcerative Colitis patients you manage, what is the percentage of patients following under each of the following types?

Numeric entry
All respondents

#	Code Label	Percent
1	Mild to moderate Ulcerative Colitis	……%
2	Moderate to severe Ulcerative Colitis	……%
3	Acute severe ulcerative colitis (ASUC) / Fulminant UC	……%
		Sum =100%

MIN: 0
MAX: 100

Q5. Among the Ulcerative Colitis patients, you manage who are moderate to severe or acute severe patients, what is the percentage of those who are controlled versus those who are un-controlled?
Please don't consider your mild-to moderate Ulcerative Colitis patients in giving the answer here (MMS= 1–3)

Numeric entry
All respondents

	Code Label	Percent of patients
1	Controlled Ulcerative Colitis Patients	....%
2	Uncontrolled Ulcerative Colitis Patients	....%
		Sum = 100%

MIN: 0
MAX: 100

Q6. Among the [insert S6. answer] Ulcerative Colitis patients you manage, please specify the percentage of naïve patients (diagnosed of UC by you) and the patients referred to you (after being initially diagnosed for UC by another specialist.)

Numeric entry

All respondents

#	Code Label	Percent of patients
1	Naïve Ulcerative Colitis patients (diagnosed by you)	………%
2	Referred Ulcerative Colitis patients (initially diagnosed by another specialist)	……...%
		Sum = 100%

MIN: 0
MAX: 100

(If Q6.2 > 0% show question Q7)

Q7. Among the Ulcerative Colitis patients who are referred to you, which specialty initially diagnosed these patients before they visit you?

Numeric entry

#	Code Label	Percent
1	General Practitioner	………%
2	IM (only show if S1=1)<br>Another IM (only show if S1=2)	………%
3	Gastroenterologist (only show if S1=2)<br>Another gastroenterologist (only show if S1=1)	………%
4	ER physician	………%
5	Hepatologist	………%
6	Surgeon	………%
7	Gynaecologists/ Obstetrician	………%
98	Others, please specify	………%
		Sum = 100%

MIN: 0
MAX: 100

B) DIAGNOSIS

Q8. Which of the following do you typically use for…

- **a. <u>Screening</u> ulcerative colitis**
- **b. <u>Confirming the diagnosis</u> of ulcerative colitis**

All respondents
Multiple answers
Randomize statements

#	Code Label	a. For screening for ulcerative colitis (MA)	b. For confirming the diagnosis of UC (MA)
1	X-ray or CT scan	☐	
2	MRE (magnetic resonance enterorrhaphy)	☐	
3	Lab tests (CBC, ESR, CRP, albumin, and liver function test)	☐	
4	Stool sample	☐	
5	Biomarkers (calprotectin and lactoferrin in stool, CRP in blood)	☐	
6	Colonoscopy/Sigmoidoscopy		☐
7	Colonoscopy/Sigmoidoscopy **<u>with biopsy</u>**		☐
8	Others, please specify	☐	☐

C) TREATMENT

Q9. What are your <u>top 3</u> treatment goals when managing your moderate to severe <u>Ulcerative Colitis</u> patients?

Numeric entry
All respondents
Randomize statements

#	Code Label	Rank
1	Endoscopic remission (Healing of mucosa)	…
2	Biochemical remission: (improved CBC, CRP, Hb% and faecal calprotectin)	…
3	Clinical Remission	…
4	Improvement lifestyle/quality of life	…
5	Avoid long term complications: hospitalization, surgery, and malignancy	…
6	Reduction of flare-up rate	…
98	Others, specify ….	…

Q10. Based on your experience, what are the top 3 barriers that prevent diagnosed Ulcerative Colitis patients from receiving treatment?
Please feel free to add other reasons if not mentioned in the list.

Maximum 3 options

#	Barriers	Rate
1	No access to healthcare system	☐
2	Fear of long-term side effects	☐
3	Patient's lack of disease awareness	☐
4	Route of administration	☐
5	Fear of colonoscopy	☐
6	Problem with referral system	☐
7	Lack of availability of the treatment	☐
8	The patient being pregnant and refusing to take treatment	☐
9	Lack of disease awareness from Obstetrician	☐
98	Other, specify ……	☐
99	Other, specify ……	☐

Q11. Please indicate the degree to which you incorporate each of the following guidelines and / or your own clinical experience into your practice for treating Ulcerative Colitis.
Kindly provide the response in percentage -

Numeric entry
All respondents

#	Code Label	Percent
1	**ECCO** (European Crohn´s and Colitis Organization)	………%
2	**AGA** (American gastroenterology association)	………%
3	**NICE** (National Institute of Health and Care Excellence)	………%
4	**ACG** (American College of Gastroenterology)	………%
5	British Society of Gastroenterology and National Health Service	………%
6	**SGA** (Saudi Gastroenterology association)	………%
7	Internal institution guidelines/protocols	………%
8	Your own clinical experience regardless the guidelines	………%
9	Other guideline, please specify	………%
		Sum = 100%

MIN: 0
MAX: 100

Q12. Based on your clinical practice, which are your most prescribed treatment classes for moderate and severe Ulcerative Colitis patients?
Please select all treatment options which are most prescribed by you in the first, second, and third treatment line for each type of Ulcerative Colitis mentioned below.

All respondents
Multiple answers per each column

#	a. Moderate to severe Ulcerative Colitis patients	1st line	2nd line	3rd line
1	5-ASA (5-aminosalicylic acid) drugs (mesalamine, Sulfasalazine)	☐	☐	☐
2	Immunomodulators (azathioprine, 6-mercaptopurine, cyclosporine, methotrexate, Cyclophosphamide etc.)	☐	☐	☐
3	Steroids	☐	☐	☐

4	TNF-α (Tumor necrosis factor-alpha) inhibitors	☐	☐	☐
5	Small molecules such as JAK inhibitors	☐	☐	☐
6	S1P (sphingosine-1-phosphate) receptor modulators	☐	☐	☐
7	IL-12/23 inhibitors (Interleukin 12/23 inhibitors)	☐	☐	☐
8	Anti-integrin	☐	☐	☐

#	b. Acute severe Ulcerative Colitis patients	1st line	2nd line	3rd line
1	5-ASA (5-aminosalicylic acid) drugs (mesalamine, Sulfasalazine)	☐	☐	☐
2	Immunomodulators (Azathioprine, 6-mercaptopurine, Cyclosporine, methotrexate, Cyclophosphamide etc.)	☐	☐	☐
3	Steroids	☐	☐	☐
4	TNF-α (Tumour necrosis factor-alpha) inhibitors	☐	☐	☐
5	Small molecules such as JAK inhibitors	☐	☐	☐
6	S1P (sphingosine-1-phosphate) receptor modulators	☐	☐	☐
7	IL-12/23 inhibitors (Interleukin 12/23 inhibitors)	☐	☐	☐
8	Anti-integrin	☐	☐	☐

c. Does the treatment choice differ in case of pregnant women with moderate to severe Ulcerative colitis?
If yes, please elaboarte how the treatment varies for a pregnant woman with moderate to severe Ulcerative colitis.

All respondents

1	**Yes**	○
2	**No**	○

……………………………………………………………………………

Show Q13 only if code 6 is not selected in Q12a in any line of treatment

Q13. Please specify the main reasons why you do not prefer prescribing S1P modulators as a treatment for moderate to severe Ulcerative Colitis patients.

Multiple answers - up to 3 answers

#	Code Label	Multiple answers up to 3 answers
1	There is not enough research supporting its use	☐
2	Not personally familiar with the treatment	☐
3	Not recommended by treatment guidelines	☐
4	Lack of availability of the medication	☐
5	Not mentioned in my hospital formulary	☐
6	Safety of the treatment	☐
7	Efficacy of the treatment	☐
98	Other (specify)	☐
99	Other (specify)	☐

Q14. Please mention the percentage distribution of your Ulcerative Colitis patients that you treated with the following options: biologics (such as TNF-α inhibitors) and small molecule agents (such as JAK inhibitors and S1P receptor modulators) in the past 3 months.

Numeric entry
All respondents

		Past 3 months	Past 3 months
#	Treatment	A. Moderate to Severe UC patients	B. Acute severe UC (ASUC)/ Fulminant UC patients
1	**Adalimumab**	..... %	..... %
2	**Infliximab**	..... %	..... %
3	**Ustekinumab**	..... %	..... %
4	**Vedolizumab**	..... %	..... %
5	**Golimumab**	..... %	..... %
6	**Tofacitinib**	..... %	..... %
7	**Upadacitinib**	..... %	..... %

8	**S1P Modulators**	..... %	..... %
9	Other (please specify...............)	..... %	..... %
		Sum up to 100%	Sum up to 100%

Q15. For how long (in months) do patients usually stay on each of the following treatments for Ulcerative Colitis treatment? Please mention the duration of stay on the treatment before switching, not the duration of the cycle of each treatment.

Numeric entry
All respondents

#	Treatment	A. Moderate to Severe UC patients
1	**Adalimumab** (Show only if Q14_1 > 0%)	.... months
2	**Infliximab** (Show only if Q14_2 > 0%)	.... months
3	**Ustekinumab** (Show only if Q14_3 > 0%)	.... months
4	**Vedolizumab** (Show only if Q14_4> 0%)	.... months
5	**Golimumab** (Show only if Q14_5 > 0%)	.... months
6	**Tofacitinib** (Show only if Q14_6 > 0%)	.... months
7	**Upadacitinib** (Show only if Q14_7> 0%)	.... months
8	**S1P Modulators** (Show only if Q14_8 > 0%)	.... months
9	Other (please specify...............) (Show only if Q14_9 > 0%)	.... months

Q16. Consider all of your Ulcerative Colitis patients who received advanced treatment such as biologics (such as TNF-α inhibitors) and small molecule agents (such as JAK inhibitors and S1P receptor modulators) in the past 3 months.
What percentage of those patients fully comply with the treatment?

All respondents
Numeric entry

Code Label	A. Moderate to Severe UC patients

Percent of Patients fully comply with advanced treatments such as biologics and small molecules	……… %

MIN: 0
MAX: 100

Q17. Consider patients who are on **advanced treatments such as biologics and small molecules such as JAK inhibitors or S1P receptor modulators**, what **criteria do** you consider before stopping the on-going treatment or switching to another treatment?

Single answer

#	Code Label	SA
1	When histological remission is not achieved	○
2	When endoscopic remission is not achieved	○
3	When symptomatic/clinical remission is not achieved	○
4	Side effects because of the current treatment	○
98	Other (Specify)…….	○

Q18. How important is each of the following factor in making treatment decisions for your Ulcerative Colitis patients?

Rate the attributes using a scale of 1 to 7, where 1= the least important and 7= the most important**.**

All respondents
Numeric entry
Randomize statements

#	Clinical Factors	Rate
1	Clinical remission	1 – 7
2	Steroid free remission	1 – 7
3	Once daily dosing	1 – 7
4	Oral form such as tablet or capsule	1 – 7
5	Rapidity of response	1 – 7
6	Appropriate for use in naïve patients	1 – 7
7	Appropriate for use in uncontrolled patients	1 – 7
8	Durable remission (data beyond 1 year)	1 – 7
9	Safety in long-term usage	1 – 7

10	Drug approved for use in multiple gastrointestinal conditions	1 – 7
11	Endoscopic remission	1 – 7

Q19. Which of the following treatment you associate the most with each of the following attributes?

All respondents
Randomize statements

#	Code Label	Most Associated (Single answer)
1	Good quality of life outcome	
2	Good safety profile	
3	Good tolerability profile	
4	Rapid onset of action	
5	Better dosage schedule	
6	Easier route of administration	
7	Better Flare-up reduction outcomes	

Data entry tables:

Code #	Treatment
1	**Adalimumab**
2	**Infliximab**
3	**Ustekinumab**
4	**Vedolizumab**
5	**Golimumab**
6	**Tofacitinib**
7	**Upadacitinib**
8	**S1P Modulators**

Q20. a. What percentage of your moderate to severe Ulcerative Colitis patients enquire or request a specific treatment?

All respondents
Numeric entry
MIN: 0
MAX: 100

	Percent
Ulcerative colitis patients who enquire or request for a specific treatment	………%

Q20.b. What percentage of your moderate to severe Ulcerative Colitis patients request for treatment in a specific formulation or route of administration?

All respondents
Numeric entry
MIN: 0
MAX: 100

	Percent
Ulcerative colitis patients who request treatment in a specific formulation	………%

Show only if 20b > 0%

Q20.c. And what is the most requested formulation (Route of Administration)?

…………………………………………………………………………

Q21. Please mention any unmet need for the currently available treatments for moderate to severe Ulcerative Colitis.

…………………………………………………………………………
…………………………………………………………………………
…………………………………………………………………………

D) TARGET MOLECULE PROFILE TESTING

Interviewer: Please show the following **information** to the physician and ask him/her to read it thoroughly.

The Kingdom of Saudi Arabia will soon have a new molecule indicated for patients with moderate to severe ulcerative colitis. Please take your time reading the following information and answer the questions based on it.

Appendix 1

Q22. What is your FIRST reaction regarding this molecule profile? How do you compare it to other molecules available in the market for Ulcerative Colitis in KSA?

All respondents

Open End

………

Q23. Based on the information you have just read, which Ulcerative Colitis patient profile do you consider most appropriate for this molecule?

All respondents

#		Multiple choice
1	**Age**	A) 18-26 years<br>B) 27-35 years<br>C) 36-43 years<br>D) 44-50 years<br>E) Over 50 years<br>F) Any UC patient above 18 years old
2	**Severity of case**	A) Moderate<br>B) Severe<br>C) Acute severe
3	**Line of treatment**	A) First line<br>B) Second line
4	**Previous treatment**	A) TNF-α inhibitor naïve<br>B) TNF-α inhibitor exposed<br>C) JAK inhibitor naïve<br>D) JAK inhibitor exposed

		E) Other, please specify

Q24. In case this molecule is available and included in the guidelines as a treatment option for the profile/s you just mentioned, what is the percentage of moderate or severe Ulcerative Colitis patients you would be willing to try this molecule in your practice?

All respondents
Numeric entry

Code label	% of patients
Percent of moderate or severe Ulcerative Colitis patients to whom I might prescribe this molecule	………%

MIN: 0
MAX: 100

Q25. Could you please share the main reason/s or rationale behind trying the new molecule in your clinical practice with [insert Q24 answer] of your moderate to severe UC patients?

All respondents
Open End (It should be a clear sentence, not general word/s, a minimum of 10-characters condition in order to accept the answer)

………
………

Q26. Based on the molecule profile just mentioned, to what extent are you willing to consider oral treatment for moderate or severe Ulcerative Colitis patients in your clinical practice in each line of treatment?

Please use a scale of 1 to 7, where "1" means <u>not at all willing</u> and "7" means <u>very willing to prescribe</u>.

All respondents
Numeric entry

Code Label	1st line	2nd Line	3rd line

Willingness to consider in clinical practice	1-7	1-7	1-7

Q27. Based on the data provided in the molecule profile, please rate each of the following statements from your point of view:

Please use a scale of 1 to 7, where "1" is means completely disagree and "7" means totally agree.

All respondents
Numeric entry

#	Code Label	Rate
1	I will include the new molecule in my treatment plan as soon as it is available in the market.	1-7
2	I will wait for more research data before I include the new molecule in my future treatment plan	1-7
3	I will wait before including the new molecule until adopted by many of my colleagues	1-7
4	I'll wait till being included in the hospital formulary	1-7
5	I'll wait till being discussed in international conferences	1-7
6	I'll wait till being included in the guidelines	1-7
7	I'll wait till being visited and discussed with the manufacturer medical liaisons/reps	1-7

Thanks for your time
